# Assessing mutant p53 in primary high-grade serous ovarian cancer using immunohistochemistry and massively parallel sequencing

**DOI:** 10.1038/srep26191

**Published:** 2016-05-18

**Authors:** Alexander J. Cole, Trisha Dwight, Anthony J. Gill, Kristie-Ann Dickson, Ying Zhu, Adele Clarkson, Gregory B. Gard, Jayne Maidens, Susan Valmadre, Roderick Clifton-Bligh, Deborah J. Marsh

**Affiliations:** 1 Hormones and Cancer Group, Kolling Institute of Medical Research, Royal North Shore Hospital, University of Sydney, NSW 2065 Australia; 2 Department of Anatomical Pathology, Royal North Shore Hospital and University of Sydney, NSW 2065 Australia; 3 Hunter New England Local Health District, Royal North Shore Hospital, University of Sydney, Australia; 4 Department of Obstetrics and Gynaecology, Royal North Shore Hospital, St Leonards, Australia; 5 Mater Private and Royal North Shore Hospitals, Sydney, NSW, Australia

## Abstract

The tumour suppressor p53 is mutated in cancer, including over 96% of high-grade serous ovarian cancer (HGSOC). Mutations cause loss of wild-type p53 function due to either gain of abnormal function of mutant p53 (mutp53), or absent to low mutp53. Massively parallel sequencing (MPS) enables increased accuracy of detection of somatic variants in heterogeneous tumours. We used MPS and immunohistochemistry (IHC) to characterise HGSOCs for *TP53* mutation and p53 expression. *TP53* mutation was identified in 94% (68/72) of HGSOCs, 62% of which were missense. Missense mutations demonstrated high p53 by IHC, as did 35% (9/26) of non-missense mutations. Low p53 was seen by IHC in 62% of HGSOC associated with non-missense mutations. Most wild-type *TP53* tumours (75%, 6/8) displayed intermediate p53 levels. The overall sensitivity of detecting a *TP53* mutation based on classification as ‘Low’, ‘Intermediate’ or ‘High’ for p53 IHC was 99%, with a specificity of 75%. We suggest p53 IHC can be used as a surrogate marker of *TP53* mutation in HGSOC; however, this will result in misclassification of a proportion of *TP53* wild-type and mutant tumours. Therapeutic targeting of mutp53 will require knowledge of both *TP53* mutations and mutp53 expression.

*TP53* is widely acknowledged as the most frequently mutated gene in human malignancy, with mutations identified in at least 50% of human cancers^1^,^2^,^3^,^4^,^5^. In the absence of cellular stress, wild-type p53 is maintained at low levels by the E3 ubiquitin ligase MDM2 that polyubiquitinates p53, marking it for proteasomal degradation. In response to stress, numerous mechanisms act to disrupt the MDM2-p53 association, resulting in stabilisation and activation of p53^6^. Activated wild-type p53 promotes processes consistent with tumour suppression, including cell cycle inhibition, apoptosis, senescence, DNA repair and autophagy, as well as processes that oppose oncogenic metabolic reprogramming [reviewed in^2^]. Mutant p53 (mutp53) results in loss of these tumour suppressive functions.

Human *TP53* maps to chromosome 17p13.1 and is composed of 19,198 nucleotides spanning eleven exons, with the coding sequence commencing in exon 2 [reviewed in^7^]. Mutations in *TP53* can occur throughout the coding sequence; however, around 80% are located in the DNA binding domain (DBD) encoded by amino acid residues 102 to 292 and encompassing six ‘hot-spot’ amino acids^7^,^8^,^9^. *TP53* mutations include single base substitutions leading to missense or nonsense mutations, deletions or insertions leading to frameshifts, in-frame deletions or insertions, as well as mutations that affect splicing. Pathogenic mutations result in loss or abrogation of wild-type p53 activity, a major mechanism likely being the inability of mutp53 to bind to response elements in DNA, preventing its function as a transcription factor. Along with the loss of wild-type function, many *TP53* mutations result in gain of an oncogenic function for the mutp53 protein. These ‘gain-of-function’ (GOF) *TP53* mutations cause mutp53 to accumulate at high levels in cells, contributing to tumorigenesis and the development of drug resistance^1^. Not all p53 mutants that lead to accumulation of p53 are proven GOF mutations; however, certain mutants such as p.Arg175His, p.Gly245Ser, p.Arg248Trp, p.Arg248Gln and p.Arg273His have been shown to actively promote tumorigenesis [reviewed in^2^]. Furthermore, a number of GOF mutations, including p.Arg175His and p.Arg273His have been linked to the development of chemoresistance^10^,^11^.

Given the broad role of p53 in malignancy, there is intense focus on its potential as a therapeutic target. Strategies include restoring normal transcriptional activity of mutp53 by restoration of wild-type protein folding using drugs such as APR-246 that are currently recruiting for Phase Ib/II clinical trials for recurrent high-grade serous ovarian cancer (HGSOC)^12^ or zinc metallochaperones^13^. Alternatively, gene replacement therapy to reintroduce wild-type p53 into mutp53 cells, most often with an adenoviral vector (Adp53), has also been shown to suppress tumour growth^14^. It is clear that success of these different approaches will be influenced by the type of *TP53* mutation present, and whether it results in accumulation of mutp53 in the cell, severe truncation of the p53 protein, or low to absent levels of p53.

HGSOC is characterised by a high frequency of *TP53* mutation. Worldwide, HGSOC is the eighth most frequent cause of cancer-related deaths in women^15^. More than 80% of women present with advanced disease (Stage III-IV), where 5 year survival is around 25%^16^. The mainstay treatment is surgical debulking followed by combinations of platinum drugs and paclitaxel; however, the majority of women relapse within two years, ultimately develop broad chemoresistance and succumb to their disease^17^.

With the advent of massively parallel sequencing (MPS) and analyses of large data sets through The Cancer Genome Atlas, *TP53* mutations have been identified at a frequency of approximately 96% in large HGSOC cohorts^18^,^19^. It has recently been suggested that 100% of HGSOC are in fact *TP53* mutant^20^. These figures are higher than first thought from reports that used less sensitive methods such as Sanger sequencing and/or only focussed on mutational ‘hot-spot’ regions of this gene^21^,^22^,^23^,^24^. *TP53* is in fact the only gene that is frequently mutated at the somatic level in HGSOC, this malignancy otherwise being characterised by low prevalence (2–6%) of recurrently mutated genes such as *BRCA1*, *BRCA2*, *RB1* and *NF1*, and a long tail of infrequently mutated genes^18^. *TP53* mutations are believed to occur early in tumorigenesis, likely in precursor lesions of ovarian cancer, highlighting the importance of mutp53 as a driver of this malignancy^25^,^26^,^27^,^28^.

Immunostaining for p53 has been used as a surrogate marker for the presence of a *TP53* mutation in HGSOC, although precise validation of this practice by comparison to detailed sequencing data has been limited, especially in the context of MPS^21^,^24^,^29^. Here, we report the use of MPS to characterise *TP53* mutations in a cohort of HGSOC in parallel with p53 immunohistochemical analyses. We note cut-offs based on our data related to percent positive p53 nuclei that should be used to increase the accuracy of p53 immunohistochemistry (IHC) as a surrogate marker of mutp53 and suggest that both p53 IHC and MPS should be considered to inform therapies targeting this protein. Further, we report *TP53* mutations in previously uncharacterised cell line models of HGSOC.

## Results

### *TP53* mutations identified using massively parallel sequencing in high- and low-grade serous ovarian cancer

**Figure 1:**
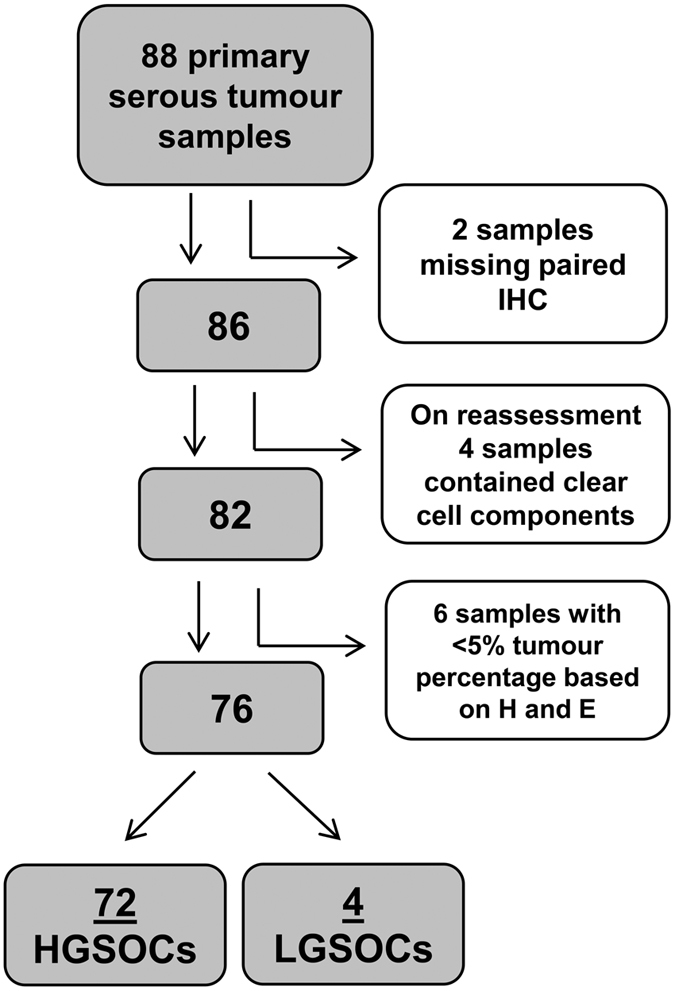
Flow diagram summarising inclusion and exclusion criteria from the primary tumour cohort. Eighty-eight primary ovarian tumours underwent MPS for *TP53*. Two samples were excluded as paired p53 IHC was unable to be obtained. Upon pathological reassessment, 4 samples were excluded due to the identification of clear cell components. A further 6 samples were excluded as they contained <5% tumour cells based on hematoxylin and eosin staining. Of the 76 remaining samples, 72 were HGSOC and 4 were LGSOC.

**Figure 2:**
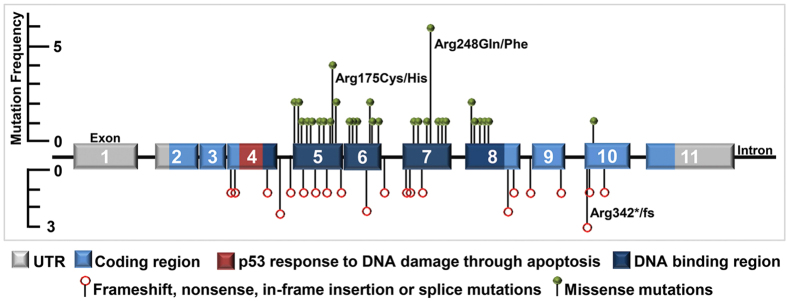
Distribution of *TP53* mutations. Grey exons mark the un-translated regions (UTR), light blue exons denote the coding regions, while dark blue exons within the coding region correlate with the DNA binding domain. The dark red region marks the p53 response to DNA damage through apoptosis area. Intronic areas are shown by the black line joining exons. All 68 mutations identified in this study are displayed as lollypop symbols, with missense mutations above the gene marked as green circles, while all ‘other’ mutation types (nonsense, frameshift, in-frame insertion and splice site) are displayed below the gene as open red circles. Mutations occurring more than once are indicated.

Depth of coverage for data generated was approximately 850 reads per amplicon per sample. A mutation in *TP53* was identified in 94% (68/72) of HGSOC. Four HGSOC reported to be wild-type for *TP53* underwent second review of archived tissue by an experienced pathologist [AJG] and remained classified as HGSOC. An additional four samples contained clear cell components and were excluded from this study (Fig. 1). The four low-grade serous ovarian cancer (LGSOC) samples contained wild-type *TP53* (Table 1; Supplementary Table S1). The majority of mutations identified (62%, 42/68) were missense, with all except one of these mutations located in the DBD of p53 (Fig. 2). The single missense mutation occurring outside of the DBD, p.Leu344Gln, was located in exon 10 of *TP53*. All missense mutations had been previously reported in The Cancer Genome Atlas ovarian study^18^ and/or the International Agency for Research on Cancer (IARC) *TP53* database (Supplementary Table S1). *TP53* mutations that had not been previously reported are indicated (Supplementary Table S1). Overall, 82% (56/68) of mutations occurred within the DBD. Frameshift, nonsense, in-frame insertion or splice mutations (38%, 26/68) were identified in exons or flanking intronic sequence between exons 4–10.

Arginine 248 (Arg248) was the most frequently mutated amino acid, with approximately 9% (6/68) of mutations (p.Arg248Gln and p.Arg248Trp) occurring at this codon, followed by Arg175 with approximately 6% (4/68) of mutations (p.Agr175His) and Arg342 with approximately 4% (3/68) of mutations (p.Arg342* and p.Arg342Glufs*2) occurring at this amino acid (Fig. 2). The frequency of mutation type we observed in our cohort was not dissimilar to that reported in recent studies^18^,^19^. The presence or location of a *TP53* mutation did not influence overall patient survival (Supplementary Fig. S1).

### Validation of *TP53* by Sanger sequencing in HGSOC

**Figure 3:**
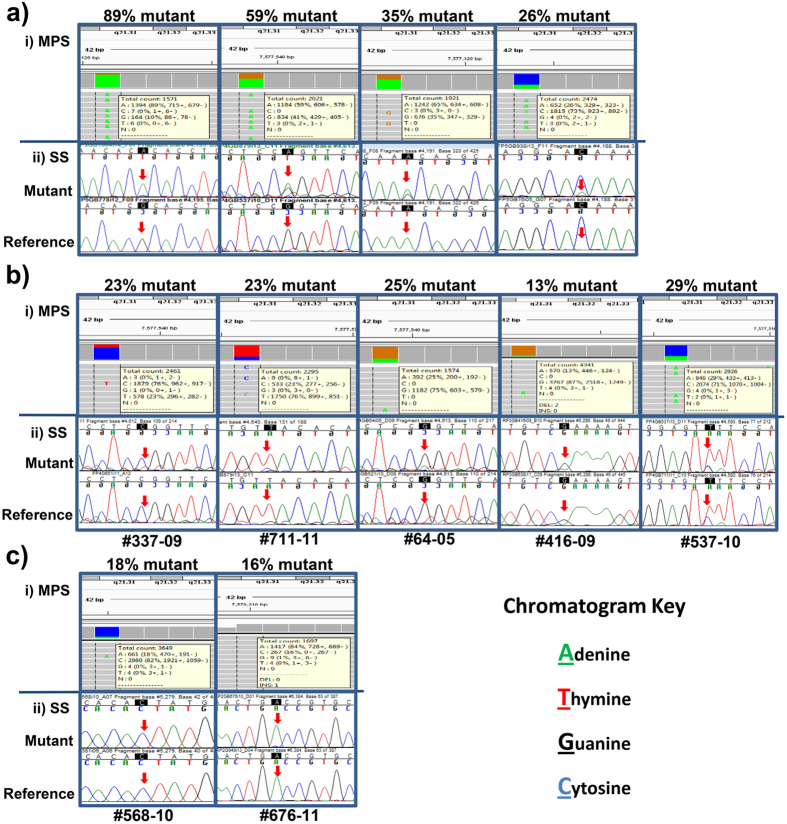
Comparison of the ability of next generation and Sanger sequencing to detect *TP53* mutations. Top panels (**a**–**c**) display MPS data viewed using IGV software (i). Lower panels display Sanger sequencing (SS) chromatograms of mutant and wild-type reference samples viewed using Sequencher software (ii). Red arrows denote presence of mutations in SS, initially discovered by MPS. (**a**) Shows four mutations with frequencies of the mutant allele between 26–89% determined by MPS that were able to be detected by SS. (**b**) Shows five mutations with frequencies of the mutant allele between 13–29%, which were initially not detected by SS; however, with the knowledge of MPS results, were identified in the chromatograms. (**c**) Shows two samples with frequencies of the mutant allele between 16–18% determined by MPS that were unable to be detected at all in SS chromatograms.

Of the 68 *TP53* mutations identified by MPS, Sanger sequencing of the identical DNA sample analysed in a blinded manner was able to identify 61 (~90%). Of the seven samples that were incorrectly determined to be *TP53* wild-type by Sanger sequencing, re-analysis with knowledge of the mutation that was identified by MPS identified small variant peaks in five samples consistent with presence of the identified mutation (Fig. 3). In the remaining two samples, no variant was detected by Sanger sequencing alone (Fig. 3). This data therefore suggests that allele frequencies of less than approximately 25% are unable to be reliably detected by Sanger sequencing.

### Correlation between *TP53* mutation status and p53 immunohistochemistry in HGSOC and LGSOC

**Figure 4:**
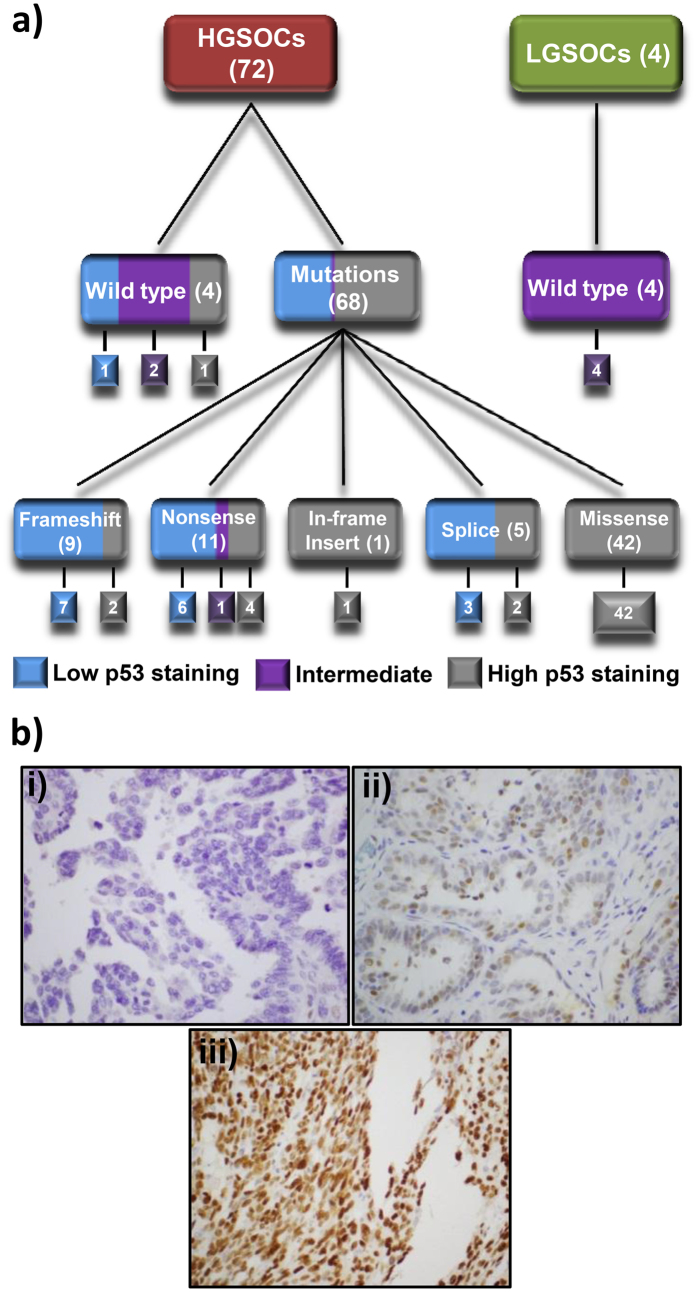
p53 immunohistochemistry and TP53 status in HGSOC and LGSOC. (**a**) Immunohistochemistry scores (‘Low’, ‘Intermediate’ or ‘High’) correlated with *TP53* mutation status. (**b**) Representative images of p53 immunohistochemistry showing (i) ‘Low’, (ii) ‘Intermediate’ and (iii) ‘High’, scores.

The overall sensitivity of detecting a *TP53* mutation based on classification of immunohistochemical staining as ‘Low’ or ‘High’ was 99% (95% Confidence Interval [CI] 91–99%), with a specificity of 75% (95% CI 36–96%). For tumours graded ‘High’ (68%; 52/76), all except one was *TP53* mutant (98%; 51/52). For tumours graded ‘Low’ (24%; 18/76), all except one were *TP53* mutant (94%; 17/18). The remaining tumours were scored ‘Intermediate’ (9%; 7/76), of which six were *TP53* wild-type (86%; 6/7) (Fig. 4a). The actual number of nuclei staining positive for p53 in the ‘Intermediate’ category ranged from 8 to 65% (Supplementary Table S2). Representative images of these staining patterns are shown in Fig. 4b and Supplementary Figs S2 and S3. The DO-7 antibody used was suitable for detecting all variants identified in our study by MPS.

**Figure 5:**
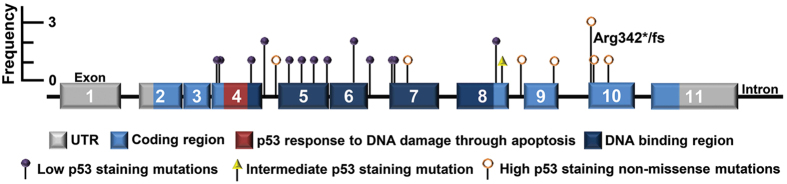
Distribution of non-missense low and high expression *TP53* mutations. Grey exons mark the un-translated regions (UTR), light blue exons denote the coding regions, while dark blue exons within the coding region correlate with the DNA binding domain. The dark red region marks the p53 response to DNA damage through apoptosis area. Intronic areas are shown by the black line joining exons. All 26 non-missense mutations identified in our cohort are displayed along *TP53*. Mutations that resulted in accumulated p53 are identified with open orange circles. The mutation that resulted in ‘Intermediate’ p53 staining is identified as a yellow triangle. Mutations that resulted in loss of p53 expression are identified as closed purple circles. Mutations occurring more than once are indicated.

All missense mutations were scored as ‘High’ for expression of the p53 protein by IHC, indicating that these mutations were associated with increased levels of mutp53. Considerable variability was observed in p53 staining levels in samples containing stop, frameshift, in-frame insertion or splice mutations (N = 26) (Fig. 4a). While the majority of samples in each non-missense mutation category were scored as ‘Low’ for p53 IHC (7 of 9 frameshift mutations, 6 of 11 nonsense mutations and 3 of 5 splice mutations), a single nonsense mutation, p.E298* (exon 8), was classified as ‘Intermediate’. The nine remaining mutations were classified as ‘High’ for p53 IHC, consisting of 2 of 9 frameshift mutations (p.Phe328fs in exon 9 and p.Arg342fs in exon 10); 4 of 11 nonsense mutations, all in exon 10 (two independent samples with p.Arg342*, p.Glu343* and p.Glu349*); 2 of 5 splice site mutations (c.376-2A > G and c.920-1G > A) (Fig. 4a); and a single in-frame insertion duplication in exon 7, c.723_724dupACCATCCACTACAACTACATGTGTAACAGTTCC. The locations of all twenty-six non-missense mutations are summarised along with their p53 IHC pattern (Fig. 5).

**Figure 6:**
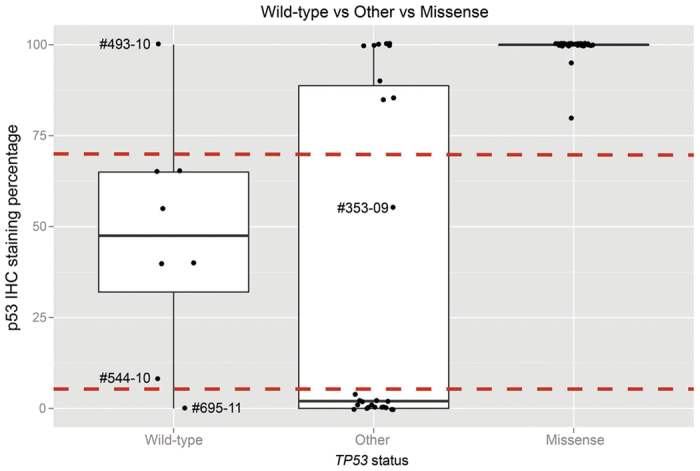
Correlation of wild-type and mutant p53 levels with *TP53* mutation status (wild-type, missense and all ‘other’ mutations). While all missense *TP53* mutants resulted in ≥70% tumour cell nuclei staining positive for p53, there was considerable variation amongst p53 staining patterns for wild-type (including both HGSOC and LGSOC) and non-missense *TP53* mutations. While the majority of wild-type *TP53* samples clustered between 8–65% tumour cell nuclei staining positive for p53 (denoted by the broken red line), one HGSOC sample demonstrated Low staining (#695-11; 0%) and one High (#493-10; 100%). Furthermore, there were two distinct clusters of non-missense mutations, with tumour cell nuclei staining positive for p53 between 80–100% and 0–4%, that could not further be discriminated by whether the mutations were nonsense, splice site, an in-frame insertion or frameshifts. Furthermore, a single nonsense mutation displayed 55% of tumour cell nuclei staining positive for p53 (#353-09).

Twelve (12/16, 75%) mutations associated with ‘Low’ expression were located within the DBD compared with two (2/9, 22%) of the mutants associated with ‘High’ expression. The mutation associated with ‘Intermediate’ expression was not located in the DBD. Furthermore, all of the mutations associated with ‘Low’ expression occurred before exon 9 compared with just 22% of the mutations associated with ‘High’ expression. All LGSOC (N = 4) were wild-type for *TP53*, and were graded ‘Intermediate’ for p53 IHC. Wild-type *TP53* HGSOC (N = 4) consisted of one sample graded as ‘High’ for p53 IHC, two ‘Intermediate’ and another as ‘Low’ (Fig. 4a). The percentage of tumour cell nuclei positive for p53 immunostaining for each mutation category, including wild-type, is depicted in Fig. 6. Whether p53 staining patterns were designated ‘Low’, ‘Intermediate’ or ‘High’ did not affect overall patient survival (Supplementary Fig. S1).

### *TP53* mutations and p53 protein expression in previously uncharacterized HGSOC and clear cell ovarian cancer cell lines

**Figure 7:**
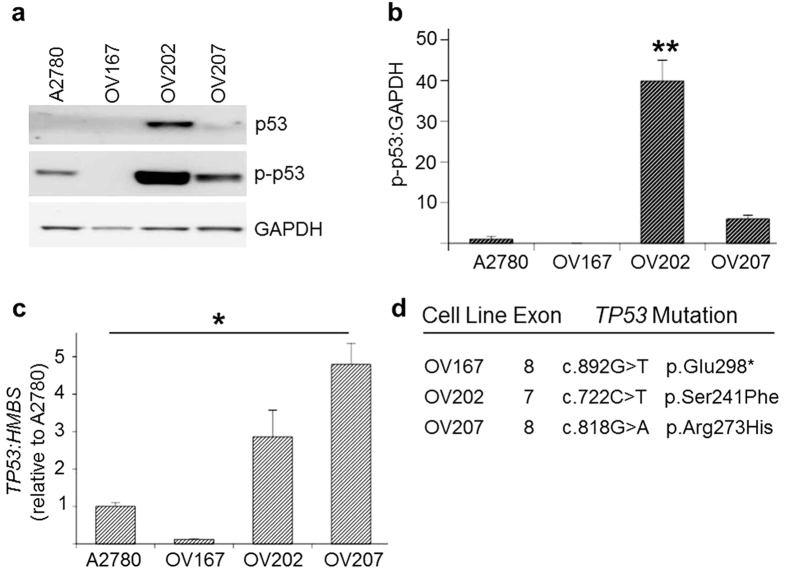
p53 protein and transcript levels in previously uncharacterized HGSOC cell lines (OV167 and OV202) and clear cell carcinoma cell line (OV207) compared to a wild-type p53 cell line (A2780). (**a**) Representative Western blots of basal levels of p53 and phosphorylated p53 (Ser15) (p-p53). (**b**) Graphical representation of protein levels of basal p-p53 in *TP53* mutant ovarian cancer cell lines relative to A2780 (N = 3, mean ± S.E.M, **P < 0.01). (**c**) Graphical representation of *TP53* transcript levels relative to the reference gene HMBS in ovarian cancer cell lines compared to A2780 (N = 3, mean ± S.E.M, *P < 0.05). (**d**) *TP53* mutations identified by our Fluidigm-MiSeq analysis pipeline in previously uncharacterised ovarian cancer cell lines.

Among the cell lines examined, Western immunoblotting showed the greatest basal p53 expression in OV202, followed by OV207. OV167 expressed a lower level of basal p53 compared to the wild-type p53 A2780 cells (Fig. 7a). Only OV202 displayed a statistically significant difference in p-p53 abundance from the other cell lines (*p* = 0.000005–0.000016; Fig. 7b). At the transcript level, all cell lines showed a significant difference between *TP53* transcripts, OV167 < A2780 < OV202 < OV207 (*p* *=* 0.000002–0.046; Fig. 7c). OV202 and OV207 were found to have the *TP53* missense mutations c.722C > T (p.Ser241Phe) and c.818G > A (p.Arg273His) respectively. OV167 had the nonsense mutation c.892G > T (p.Glu298*) (Fig. 7d). Further, immunostaining of a cell pellet of OV167 showed an “Intermediate” staining pattern similar to the tumour sample #353-09 shown to have the identical mutation (Supplementary Fig. S4). The mutant allele frequency for each of these cell lines was ≥99% as determined by MPS and our analysis pipeline.

## Discussion

Mutation of *TP53* is pervasive in HGSOC; however, whether these mutations lead to accumulation of mutp53 and possible dominant-negative and/or gain-of-function effects, or low to absent levels of mutp53 is dependent upon individual mutations. Using MPS, we identified 94% (68/72) of HGSOC with *TP53* mutation and 100% (4/4) of LGSOC were wild-type for *TP53*, similar to recent studies^18^,^19^,^24^. Pathological reassessment of the four *TP53* wild-type HGSOC did not change the initial diagnosis of HGSOC. When correlated with p53 IHC data for the same specimens, we showed an overall sensitivity of 99% for detecting a *TP53* mutation based on an IHC score of ≤5% or ≥70% percent p53 positive nuclei; however, this test showed a lower specificity of 75%. In a cohort of HGSOC where the expectation is that the vast majority of samples will be *TP53* mutant, p53 IHC may be considered an acceptable surrogate marker for *TP53* mutation. In tumour types where the frequency of somatic *TP53* mutation is expected to be much lower, the lower specificity of this test may impact on its value as a surrogate marker of *TP53* mutation.

There is general agreement in the literature that both high and low, or entirely absent, p53 expression is correlated with mutant p53, while expression levels between these two extremes are thought to represent wild-type p53. In studies of HGSOC, high levels of p53 positive nuclei indicative of mutp53 have been described as >30%^30^, >50%^31^,^32^ and ≥60%^21^, although it is noted in one report that the majority of high expressors showed >85% p53 positive nuclei^31^. In the current study, the cut-off for high p53 expression was ≥70%. Reduction of this cut-off to lower levels previously published would have resulted in misclassification of several wild-type *TP53* tumours as mutant and lowered the overall specificity of this test. A lower cut-off for high expression would; however, have correctly classified tumour #353-09 that displayed 55% p53 positive nuclei and was misclassified under our criteria.

Low levels of p53 expression correlated with the presence of a mutation have been described as completely negative^21^,^31^, although tumours with wild-type *TP53* have also been reported to have low p53 levels, ≤10% positive nuclei^21^, potentially complicating clear discrimination between mutant and wild-type p53. In the current study, 10 tumours showed complete absence of p53, one of which was wild-type. A further 7 tumours, all *TP53* mutant, showed <5% positive p53 nuclei (1–4%). We suggest that even with careful optimisation of p53 immunohistochemistry, this lower range of p53 expression will pose problematical for the discrimination of all wild-type from mutp53 tumours.

The majority of *TP53* mutations identified in this study (82%; 56/68) were located in the p53 DBD. Hot-spot codons for mutation included p.Arg248 and p.Arg175 residing within the p53 DBD, that have previously been recognized as *TP53* mutational hot-spots in human malignancy^2^, as well as p.Arg342. No differences in overall survival in HGSOC were observed that could be correlated with presence or absence of a *TP53* mutation, mutation type or location; however, we could not exclude the possibility that specific mutations, or mutations at certain amino acids, may in fact influence patient survival.

Sanger sequencing of samples in which a mutation had been identified by MPS proved to be less efficient at detecting mutations, failing to identify approximately 10% of the mutations that were identified by MPS when analyses were performed in a blinded fashion. The lower sensitivity of this technique in comparison to MPS may at least partly explain the lower frequency of *TP53* mutations in HGSOC reported in earlier studies^21^,^23^. This will have impacted on the accuracy of previous studies where attempts have been made to correlate *TP53* mutation and p53 IHC^21^. In the current study, Sanger sequencing was unable to robustly identify mutations where the frequency of the mutated allele determined by MPS was less than 25%. This limit of detection is supported by other studies^33^,^34^.

The majority (62%) of mutations identified in HGSOC were missense, all occurring in the p53 DBD with the exception of p.Leu344Gln in exon 10. All tumours with missense mutations were scored as ‘High’ by p53 IHC, consistent with accumulation of mutp53. This data suggests that p53 IHC is a strong surrogate marker of *TP53* missense mutation; however, it was not specific for this class of mutation. Four mutations in exon 10 classified as nonsense (two tumours with p.Arg342*, p.Glu343* and p.Glu349*) and two with frameshifts (p.Phe328Hisfs*7 in exon 9 and p.Arg342Glufs*2 in exon 10) also resulted in accumulation of mutp53 and a score of ‘High’ for IHC. All of the nonsense and frameshift mutations associated with high levels of mutp53 would be predicted to truncate p53 towards its carboxyl-terminus. Splice site mutations (c.376-2A > G in intron 4 and c.920-1G > A in intron 8), as well as an in-frame insertion of a duplication in exon 7, also resulted in accumulation of mutp53 and a ‘High’ p53 IHC score. The remaining nonsense, frameshift and splice site mutations resulted in ‘Low’ p53 IHC scores, with the exception of a single nonsense mutation, p.Glu348* in exon 8, that demonstrated an ‘Intermediate’ p53 staining pattern. Two distinct clusters of non-missense mutations in the context of cellular accumulation of mutp53 were present in this study; however, they could not be clearly defined based on mutation type or location.

Four HGSOC were identified as wild-type for *TP53*. These fresh frozen specimens contained between 30–80% tumour cells. In the absence of mutation or stimulus as the result of DNA damage, wild-type p53 would be expected to be present at low levels in the cell^6^. Of the HGSOCs that were wild-type for *TP53*, one showed ‘High’ p53 IHC, two ‘Intermediate’ and one ‘Low’. As expected, the four LGSOCs were *TP53* wild-type, and all showed ‘Intermediate’ staining for p53 IHC. It is possible that the wild-type *TP53* tumours demonstrating ‘High’ or ‘Low’ levels of accumulated p53 may have other abnormalities, such as amplification of *MDM2* or *MDM4* that has previously been associated with some, but not all, *TP53* wild-type HGSOC^29^. The literature remains conflicted as to whether differing levels of mutp53 are associated with survival^24^,^29^,^31^,^32^,^35^,^36^,^37^; however, we did not find any association between overall survival and levels of mutp53 expression in this study (Supplementary Fig. S1).

Recent genomic assessment of cell line models of HGSOC has identified cell lines believed to be stronger models for studying this cancer based on their genomic similarity to primary tumours^38^,^39^. All cell lines ranking as strong models for HGSOC were *TP53* mutant. Two HGSOC cell lines not included in these published studies, OV167 and OV202, contained a nonsense and missense *TP53* mutation respectively. A third cell line established from a clear cell adenocarcinoma, OV207, also contained a missense *TP53* mutation. While our reporting of *TP53* mutation status in these cell lines reflects only part of the extensive genomic analyses of cell lines performed by other studies^38^,^39^, it does support the likelihood that these are suitable cell line models for studying this malignancy.

In summary, we suggest that p53 IHC may be used with care as a surrogate marker of *TP53* mutation by designating specimens demonstrating ‘Intermediate’ p53 staining as most likely p53 wild-type. However, detailed knowledge of mutation effect determined by the combination of MPS, mutation location in *TP53* and levels of p53 expression may be particularly important when considering options for therapeutic targeting of mutant and wild-type p53 that are currently in development^40^. The success of therapeutic targeting of mutant p53 will in large part depend on knowledge of both the level of p53 expression and the exact nature of the mutation. While this study has focussed on HGSOC, combining MPS of *TP53* with p53 IHC should also be considered when investigating different therapeutic strategies for targeting p53 in other human malignancies. Sensitivities and specificities of these tests should be determined for other malignancies that demonstrate a range of *TP53* mutation frequencies.

## Materials and Methods

### Tumour Samples

Our cohort consisted of 88 gynecological tumours (serous ovarian, fallopian tube and peritoneal carcinomas) collected from 2004 to 2014 from three hospitals (Royal North Shore Hospital, North Shore Private and The Mater Hospital-North Sydney, Sydney, Australia). Seventy-two HGSOC and four low grade serous tumours (LGSOC) were included in the final analysis. Rationale for specimen exclusion is described in Fig. 1. Eighty-two percent (59/72) of HGSOCs were at an advanced stage, classified as Stage III or IV. Survival and other clinical data were available for the majority of patients (Supplementary Table S3). Survival times were defined as time from date of surgery to last follow up or death as recorded in hospital medical records, doctors’ rooms and publicly available death notices. All snap frozen tumour samples were collected and stored in the Kolling Institute of Medical Research (KIMR) Gynecological Tumour Bank. Matched formalin fixed paraffin embedded samples were obtained from one of two diagnostic pathology laboratories (Pacific Laboratory Medicine Services, PaLMS or Douglass Hanly Moir Pathology, Sydney, Australia). Ethical approval for this project was obtained from the Northern Sydney Local Health District Human Research Ethics Committee (Protocol: 108–243 M). Written informed consent was obtained from all subjects. All methods were carried out in accordance with the approved guidelines. All tumour tissue was reviewed by a pathologist [AJG] to confirm diagnosis, histological grade and pathological stage. Sequential sections were ascertained from frozen tumours for determination of percent tumour cells following hematoxylin and eosin staining, and for DNA extraction (Supplementary Table S1). Tumours containing at least 5% of tumour cells were selected for this study.

DNA was prepared from approximately 30 mg fresh frozen tumour tissue, homogenised in phosphate buffered saline using a Retsch MM 301 Mixer Mill (MEP Instruments Pty. Ltd., NSW, Australia). Tissue was shaken three times for 90 seconds at the highest frequency until liquefied. Protein was digested overnight at 56 °C with proteinase K (Qiagen Pty Ltd, Chadstone, VIC, Australia). DNA was extracted using the DNeasy Blood and Tissue Kit and QIAcube (Qiagen Pty Ltd, Chadstone, VIC, Australia). DNA concentration was determined by Qubit Fluorometric Quantitation (Life Technologies Australia Pty. Ltd., Mulgrave, VIC, Australia) and 260:280 and 260:230 ratios determined using a NanoDrop ND-1000 spectrophotometer (Thermo Fisher Scientific Australia, Scoresby, VIC, Australia).

### Ovarian Cancer Cell Lines

Three ovarian cancer cell lines that had not been previously characterised for *TP53* status, HGSOC cell lines OV167 and OV202, and the clear cell adenocarcinoma line OV207, were analysed (cell lines were kindly provided by Drs C. Conover and K. Kalli, Mayo Center, Rochester, MN, USA)^41^. All cell lines were cultured in RPMI 1640 (Gibco, Life Technologies Australia Pty Ltd, Mulgrave, VIC, Australia) supplemented with 10% (volume/volume) fetal bovine serum (SAFC Bioscience, Brooklyn, VIC, Australia) at 37 °C with 5% CO_2_ as previously described^42^. Cells were grown to 80% confluence prior to harvesting for DNA extraction. DNA was extracted using the DNeasy Blood and Tissue Kit and QIAcube (Qiagen Pty Ltd, Mulgrave, VIC, Australia).

Cell lines were authenticated by short tandem repeat profiling using the AMPFLSTR® Identifiler® PCR Amplification Kit (Applied Biosystems), a 16 loci (15 STR loci plus Amelogenin) STR multiplex kit performed by CellBank Australia (Westmead, New South Wales, Australia). Authentication profiles are available for OV202 and OV167^42^ and OV207 (Supplementary Table S4).

Approximately 3 × 10^6^ of the *TP53* missense HGSOC cell line OVCAR-3, p53 null cell line SK-OV-3, *TP53* wild-type line A2780 and *TP53* nonsense mutation line OV167 were harvested, washed twice with PBS and centrifuged at 400 RCF for 3 minutes. Supernatant was discarded, cells resuspended in 100 μl of 1% agarose (made up in PBS) and left at room temperature for 5 minutes for the agarose to set. Cell plugs were fixed using 10% formalin for 6 h before being dehydrated by changes in graded alcohol and xylene and embedded in a paraffin block. Cell lines underwent immunohistochemistry for p53 as described.

### Western Immunoblotting and Gene Expression

Cells were lysed in RIPA buffer (Sigma-Aldrich, Castle Hill, NSW, Australia) containing Protease Inhibitor Cocktail (Sigma-Aldrich), phenylmethylsulfonyl fluoride (PMSF; Sigma-Aldrich) and the phosphate inhibitor cocktail PhosStop (Roche Diagnostics, Castle Hill, NSW, Australia). Thirty μg of total protein was added to 4 × NuPAGE® LDS Sample Buffer (Thermo Fisher Scientific, Waltham, MA, USA), followed by heating at 70 °C for 10 minutes. Cell lysates were electrophoresed on 4–12% Bis-Tris gels (Life Technologies, Thornton, NSW, Australia) at 180 V for one hour followed by transfer to a nitrocellulose membrane (Amersham™ Protran® Supported Western Blotting Membrane, GE Healthcare, Sigma-Aldrich) using a wet transfer system at 100 V for 1.5 hours (Bio-Rad Laboratories, Gladesville, NSW, Australia). Membranes were blocked with 5% skim milk for one hour at ambient temperature, and probed overnight at 4 °C with primary antibody (p53 [1C12] mouse mAb, Phospho-p53(Ser15) rabbit polyclonal antibody, or GAPDH [14C10] rabbit mAb; all from Cell Signaling Technology, Danvers, MA, USA). Membranes were probed with peroxidase labeled secondary antibodies (anti-rabbit IgG HRP-linked whole antibody or anti-mouse IgG-HRP, both from GE Healthcare, VWR International, Murarrie, Queensland, Australia) for one hour. Chemiluminescent signal was detected by SuperSignal ECL Dura reagent (Pierce, Rockford, IL, USA) and visualised using the Fujifilm LAS-4000 imaging system (Berthold Australia Pty. Ltd., Bundoora, VIC, Australia). Quantitation was undertaken using Multi Gauge 3.0 (Fujifilm Australia Pty. Ltd., Brookvale, NSW, Australia).

Total RNA was extracted from all cell lines using the miRNeasy Mini Kit (Qiagen, Doncaster, VIC, Australia) and automated using a QIAcube (Qiagen). 500 μg RNA was reverse transcribed using Superscript III reverse transcriptase (Life Technologies). Quantitative real-time RT-PCR was performed in triplicate using a TaqMan Gene Expression Assay for p53 (Hs01034249_m1; Life Technologies) and an endogenous reference gene (hydroxymethylbilane synthase (HMBS); Hs00609297_m1, Life Technologies), using iTaq Universal Probes Supermix (Bio-Rad Laboratories) on a 7900HT Fast Real-Time PCR System (Applied Biosystems). Reagents were aliquoted using an epMotion 5070 robot (Eppendorf South Pacific Pty Ltd., North Ryde, NSW, Australia).

### p53 Immunohistochemistry

Immunohistochemistry for p53 was performed on formalin fixed paraffin embedded tissue sections using a commercially available mouse monoclonal anti-human antibody (Protein Clone DO-7, cat. #M7001, Dako, CA, USA) at a dilution of 1 in 50. Staining was performed on a representative whole section using an automated staining platform - the Leica BOND-III™ autostainer (Leica Biosystems, Mount Waverley, Victoria, Australia). Heat Induced Epitope Retrieval (HIER) was performed for 30 minutes at 97 °C in the manufacturer’s acidic retrieval solution (ER1: VBS part no: AR9961). Stained slides were examined by an experienced surgical pathologist [AJG] who was blinded to molecular data. The percentage of cells showing positive nuclear staining was estimated and reported in three categories: ≥70% positively stained nuclei (High); >5% and <70% stained nuclei (Intermediate); ≤5% positively stained nuclei (Low).

### *TP53* genomic PCR with the Fluidigm Access Array™ System and Massively Parallel Sequencing (MPS)

DNA from tumour and cell line samples were processed for MPS using the Access Array™ BRCA1/BRCA2/TP53 Target-Specific Panel (Fluidigm, South San Francisco, CA, USA). The 48.48 Access Array™ integrated fluidic circuits (IFC) was used, including target specific primers containing a common sequence tag (CS1 or CS2) and Illumina adaptors PE1 and PE2. A sample specific barcode was located on the reverse sequence (PE1_CS1 Forward Primer, 5′-AATGATACGGCGACCACCGAGATCTACACTGACGACATGGTTCTACA-3′, 47 bp; PE2_BC_CS2 Reverse primer, 5′-CAAGCAGAAGACGGCATACGAGAT[sample specific barcode]TACGGTAGCAGAGACTTGGTCT-3′, 56 bp). This array enabled 92% coverage of *TP53* using 16 primer pairs generating amplicons of between 191–209 base pairs. Five μl of DNA (50 ng/μl) was added to the array and run on the Fluidigm Biomark HD™ Real-Time PCR fluidics system according to the manufacturer’s guidelines and performed by the Ramaciotti Centre for Genomics (University of New South Wales, Randwick, Australia). Amplicon libraries were pooled and a single MPS run was performed on a MiSeq platform using Miseq Control Software (MCS) version 2.4.1 (Illumina Inc., San Siego, CA, USA).

Sequencing data was received in FASTQ file format. FASTQ files were aligned to the human genome (hg19) using Burrows-Wheeler Aligner (BWA) and variant calling was performed using the Genome Analysis Toolkit (GATK), according to GATK best practice (https://www.broadinstitute.org/gatk/guide/best-practices). Annotation of variant calls was performed using ANNOVAR, version 2013 Jul^43^, while visualisation of data was performed with the Broad Institute’s Integrative Genomics Viewer (IGV, v2.3, ww.broadinstitute.org).

Each sample summary was imported into Excel and filtered to display *TP53* exonic and splice mutations, excluding mutations identified in intronic regions. Filtering criteria were applied to remove reads with a quality (QUAL) score less than 100. Mutations were further filtered based on their frequency in the 1000 Genome Database^44^. If a particular variant occurred at a frequency greater than 10% in this database, the mutation was deemed to be non-deleterious and excluded from our analysis. Lastly, mutations were filtered based on SIFT scores, an *in silico* tool for predicting functional effects of a mutation on the associated protein^45^. Variants predicted to be tolerated were excluded. All remaining variants were considered deleterious or did not have a SIFT score and were visualized using IGV. The allele frequency of each mutation was recorded upon verification of the mutation *via* IGV. Our analysis pipeline is summarised in Supplementary Fig. S5.

### Sanger Sequencing to validate *TP53* mutations

All samples in which a *TP53* mutation was identified by MPS underwent Sanger sequencing using the identical DNA. Primers and PCR conditions used were those recommended by the International Agency for Research on Cancer (IARC) *TP53* database (http://p53.iarc.fr/; Supplementary Table S5). PCR products were purified using a DNA Clean & Concentrator™ Kit (Zymo Research Corporation, Irvine, CA, USA) and commercial sequencing undertaken by the Australian Genome Research Facility (AGRF; Sydney, NSW, Australia). *TP53* variants were annotated against the human *TP53* reference genomic sequence NC_000017.10 using Sequencher DNA Sequence Analysis Software version 4.9 (Gene Codes Corporation, Ann Arbor, MI, USA).

### Statistical Analyses

Specificity and sensitivity of the association between p53 IHC score and the presence or absence of a *TP53* mutation were analysed using the VassarStats Statistical Computation website (http://www.vassarstats.net/). Western blot and qRT-PCR data from analyses of ovarian cancer cell lines are expressed as the mean ± S.E.M. from at least three independent experiments. Statistical significance was determined by one-way ANOVA. Kaplan-Meier survival analyses compared by the Log Rank (Mantel-Cox) test were undertaken to determine whether the type or location of *TP53* mutation, or p53 staining pattern (graded as ‘High’, ‘Intermediate’ or ‘Low’) influenced overall survival in HGSOC. *P* < 0.05 was considered statistically significant. With the exception of specificity and sensitivity calculations above, all other data analyses were undertaken using IBM SPSS software version 21.0 (SPSS Australasia Pty Ltd., Chatswood, NSW, Australia).

## Additional Information

**How to cite this article**: Cole, A. J. *et al.* Assessing mutant p53 in primary high-grade serous ovarian cancer using immunohistochemistry and massively parallel sequencing. *Sci. Rep.* **6**, 26191; doi: 10.1038/srep26191 (2016).

## Supplementary Material

Supplementary Information

## Figures and Tables

**Table 1:** *TP53* mutations and immunohistochemical classification for high- and low-grade serous ovarian cancer.

	Frequency	High IHC	Low IHC	Intermediate IHC
High-grade serous ovarian cancer				
Frameshift	9	2 (22%)	7 (78%)	0 (0%)
Nonsense	11	4 (36%)	6 (55%)	1 (9%)
Splice	5	2 (40%)	3 (60%)	0 (0%)
Missense	42	42 (100%)	0 (0%)	0 (0%)
In-frame insertion	1	1 (100%)	0 (0%)	0 (0%)
Wild-type	4	1 (25%)	1 (25%)	2 (50%)
Total Mutations	68 (94%)			
Total Samples	72			
Low-grade serous ovarian cancer				
Wild-type	4	0 (0%)	0 (0%)	4 (100%)
Total Mutations	0			
Total Samples	4			

Tumours with ≥70% positive p53 nuclei were categorized as ‘High’ IHC. Tumours with >5% and <70% positive p53 nuclei were categorized as ‘Intermediate’ IHC, while samples with ≤ 5% positive p53 nuclei were categorized as ‘Low’ IHC.
