# Supplementary material for: Assessing mutant p53 in primary high-grade serous ovarian cancer using immunohistochemistry and massively parallel sequencing
Source: Sci Rep. 2016 May 18;6:26191. doi: 10.1038/srep26191 (PMC4870633; doi:10.1038/srep26191)
Supplement: Supplementary Information [file srep26191-s1.pdf]

Assessing mutant p53 in primary high-grade serous ovarian cancer using immunohistochemistry and massively parallel sequencing

Alexander J Cole, Trisha Dwight, Anthony J Gill, Kristie-Ann Dickson, Ying Zhu, Adele Clarkson, Gregory B Gard, Jayne Maidens, Susan Valmadre, Roderick Clifton-Bligh and Deborah J Marsh

Supplementary Table S1. Massively Parallel Sequencing data of all samples identified to contain a *TP53* mutation; cDNA aligned to the *TP53* sequence NM_000546. Sample which were unable to be confirmed by Sanger sequencing are labelled with ^. **a)** Displays data from the sixty-eight HGSOCs containing a *TP53* mutation. **b)** Displays data from the three ovarian cancer cell lines OV202, OV207 and OV167.

a)

Sample ID	Genomic position (chr:start-end)	Reference allele (%)	Variant allele (%)	Exon	cDNA change	Protein effect	Mutation type	Tumor reference allele read count	Tumor variant allele read count	Tumor variant allele ratio	LOH ≥70 %	No. of cases with variant	Functional classification (SIFT)	Database Presence (IARC)	Percent Tumour composition
10-04	17:7579530-7579530	-	T (41%)	4	c.156_157insA	p.Trp53Metfs*3	Frameshift	1217	824	0.41	No	1	-	No	70
14-04	17:7577550-7577550	C (20%)	A (80%)	7	c.731G>T	p.Gly244Val	Missense	608	2439	0.80	Loss of WT	1	Deleterious	Yes	80
18-04	17:7577536-7577536	T (9%)	C (91%)	7	c.745A>G	p.Arg249Gly	Missense	221	2159	0.91	Loss of WT	1	Deleterious	Yes	80
40-05	17:7578556-7578556	T (34%)	C (66%)	Intron 4	c.376-2A>G	-	Splice	388	758	0.66	No	1	-	Yes	80
47-05	17:7573996-7573996	A (32%)	T (68%)	10	c.1031T>A	p.Leu344Gln	Missense	506	1075	0.68	No	1	Deleterious	Yes	60
64-05	17:7577539-7577539	G (75%)	A (25%)	7	c.742C>T	p.Arg248Trp	Missense	1182	392	0.25	No	2	Deleterious	Yes	60^
74-05	17:7577599-7577599	-	A (90%)	7	c.681_682insT	p.Asp228fs*1	Frameshift	909	824	0.90	Loss of WT	1	-	Yes	80
76-05	17:7577100-7577100	T (37%)	C (63%)	8	c.838A>G	p.Arg280Gly	Missense	807	1356	0.63	No	1	Deleterious	Yes	10
105-06	17:7578406-7578406	C (58%)	T (42%)	5	c.524G>A	p.Arg175His	Missense	1640	1166	0.42	No	3	Deleterious	Yes	40
106-06	17:7578402-7578402	G (49%)	C (51%)	5	c.528C>G	p.Cys176Trp	Missense	1392	1444	0.51	No	1	Deleterious	Yes	70
109-06	17:7574003-7574003	G (27%)	A (73%)	10	c.1024C>T	p.Arg342*	Nonsense	457	1219	0.73	Loss of WT	2	NA	Yes	50
94-06	17:7578554-7578554	A (24%)	C (76%)	5	c.376T>G	p.Try126DAsp	Missense	223	709	0.76	Loss of WT	1	Deleterious	Yes	80
99-06	17:7578527-7578527	A (18%)	C (82%)	5	c.403T>G	p.Cys135Gly	Missense	410	2251	0.82	Loss of WT	1	Deleterious	Yes	60
123-07	17:7577538-7577538	C (37%)	T (63%)	7	c.743G>A	p. Arg248Gln	Missense	839	1441	0.63	No	4	Deleterious	Yes	70
133-07	17:7578208-7578208	T (46%)	C (54%)	6	c.641A>G	p.His214Arg	Missense	1912	2290	0.54	No	2	Deleterious	Yes	70
156-07	17:7578272-7578272	G (46%)	A (54%)	6	c.577C>T	p.His193Tyr	Missense	1580	1820	0.54	No	1	Deleterious	Yes	60

157-07	17:7578265-7578265	A (67%)	G (33%)	6	c.584T>C	p.Ile195Thr	Missense	1746	868	0.33	No	1	Deleterious	Yes	15
198-08	17:7577058-7577058	C (57%)	A (43%)	8	c.880G>T	p.Glu294*	Nonsense	1098	826	0.43	No	2	NA	Yes	10
206-08	17:7577117-7577117	A (65%)	G (35%)	8	c.821T>C	p.Val274Ala	Missense	1242	676	0.35	No	1	Deleterious	Yes	50
230-08	17:7578431-7578431	-	T (24%)	5	c.499dupA	p.Gln167Thrfs*13	Frameshift	3747	884	0.24	No	1	-	No	50
237-08	17:7578208-7578208	T (58%)	C (42%)	6	c.641A>G	p.His214Arg	Missense	2382	1755	0.42	No	2	Deleterious	Yes	30
309-09	17:7573982-7573982	C (11%)	A (89%)	10	c.1045G>T	p.Glu349*	Nonsense	166	1324	0.89	Loss of WT	1	NA	Yes	40
337-09	17:7577538-7577538	C (76%)	T (24%)	7	c.743G>A	p.Arg248Gln	Missense	1879	578	0.24	No	4	Deleterious	Yes	30^
353-09	17:7577046-7577046	C (72%)	A (28%)	8	c.892G>T	p.Glu298*	Nonsense	1611	623	0.28	No	1	NA	Yes	7
381-09	17:7579311-7579311	C (79%)	T (21%)	Intron 4	c.375+1G>A	-	Splice	1224	320	0.21	No	1	-	Yes	15
416-09	17:7578212-7578212	G (87%)	A (13%)	6	c.637C>T	p.Arg213*	Nonsense	3767	570	0.13	No	2	NA	Yes	10^
466-10	17:7574003-7574003	G 59%)	A (41%)	10	c.1024C>T	p.Arg342*	Nonsense	1231	856	0.41	No	2	NA	Yes	30
471-10	17:7578404-7578404	A (43%)	T (57%)	5	c.526T>A	p.Cys176Ser	Missense	1629	2158	0.57	No	1	Deleterious	Yes	80
472-10	17:7578440-7578440	T (39%)	C (61%)	5	c.490A>G	p.Lys164Glu	Missense	1454	2239	0.61	No	1	Deleterious	Yes	80
490-10	17:7576862-7576862	-	A (38%)	9	c.983_984insT	p.Phe328Hisfs*7	Frameshift	1109	423	0.38	No	1	-	No	15
497-10	17:7578212-7578212	G (52%)	A (48%)	6	c.637C>T	p.Arg213*	Nonsense	2252	2059	0.48	No	2	NA	Yes	70
521-10	17:7578176-7578176	C (30%)	T (70%)	Intron 6	c.672+1G>A	-	Splice	749	1716	0.70	Loss of WT	1	-	Yes	80
531-10	17:7577538-7577538	C (21%)	T (79%)	7	c.743G>A	p.Arg248Gln	Missense	461	1748	0.79	Loss of WT	4	Deleterious	Yes	80
537-10	17:75775067577506	C (71%)	A (29%)	7	c.775G>T	p.Asp259Try	Missense	2074	846	0.29	No	1	Deleterious	Yes	50^
543-10	17:7577106-7577106	G (19%)	A (81%)	8	c.832C>T	p.Pro278Ser	Missense	514	2248	0.81	Loss of WT	1	Deleterious	Yes	60
565-10	17:7576927-7576927	C (64%)	T (36%)	Intron 8	c.920-1G>A	-	Splice	1159	660	0.36	No	1	-	Yes	30
568-10	17:7578205-7578205	C (82%)	A (18%)	6	c.644G>T	p.Ser215Ile	Missense	2980	661	0.18	No	1	Deleterious	Yes	20^
586-11	17:7578398-7578398	-	C (78%)	5	c.531_532insG	p.His178Alafs*2	Frameshift	2018	1571	0.78	Loss of WT	1	-	No	85
612-11	17:7578406-7578406	C (41%)	T (59%)	5	c.524G>A	p.Arg175His	Missense	1012	1433	0.59	No	3	Deleterious	Yes	85

614-11	17:7578449-7578449	C (20%)	T (80%)	5	c.481G>A	p.Ala161Thr	Missense	354	1417	0.80	Loss of WT	1	Deleterious	Yes	60
630-11	17:7574000-7574000	C (24%)	A (76%)	10	c.1027G>T	p.Glu343*	Nonsense	358	1125	0.76	Loss of WT	1	NA	Yes	35
631-11	17:7578406-7578406	C (53%)	T (47%)	5	c.524G>A	p.Arg175His	Missense	1707	1527	0.47	No	3	Deleterious	Yes	85
634-11	17:7578190-7578190	T (54%)	C (46%)	6	c.659A>G	p.Try220Cys	Missense	1553	1314	0.46	No	1	Deleterious	Yes	85
638-11	17:7578485-7578485	A (17%)	- (83%)	5	c.445delT	p.Ser149Profs*20	Frameshift	456	2286	0.83	Loss of WT	1	-	Yes	90
651-11	17:7577594-7577594	A (13%)	T (87%)	7	c.687T>A	p.Cys229*	Nonsense	133	907	0.87	Loss of WT	1	NA	Yes	40
666-11	17:7577509-7577509	C (21%)	T (79%)	7	c.772G>A	p.Glu258Lys	Missense	562	2084	0.79	Loss of WT	1	Deleterious	Yes	70
676-11	17:7579310-7579310	A (84%)	C (16%)	Intron 4	c.375+2T>G	-	Splice	1417	268	0.16	No	1	-	Yes	5^
679-11	17:7577574-7577574	T (14%)	C (86%)	7	c.707A>G	p.Try236Cys	Missense	270	1720	0.86	Loss of WT	1	Deleterious	Yes	80
694-11	17:7578253-7578254	C (34%) C (34%)	A (66%) A (66%)	6	c.[595G>T];[596G>T]	p.Gly199Leu	Missense	1425	2739	0.66	No	1	Deleterious	Yes	50
702-11	17:7578413-7578413	C (52%)	A (48%)	5	c.517G>T	p.Val173Leu	Missense	1479	1369	0.48	No	1	Deleterious	Yes	50
711-11	17:7577566-7577566	T (76%)	C (23%)	7	c.715A>G	p.Asn239Asp	Missense	1750	533	0.23	No	1	Deleterious	Yes	15^
764-12	17:7579341-7579345	AATGC (68%)	- (32%)	4	c.342_346del	p.Ser116Phefs*32	Frameshift	1821	862	0.32	No	1	-	No	20
767-12	17:7578446-7578447	TG (48%)	- (52%)	5	c.483_484del	p.Ala161Leufs*17	Frameshift	900	964	0.52	No	1	-	No	60
778-12	17:7574003-7574003	G (64%)	- (36%)	10	c.1024delC	p.Arg342Glufs*2	Frameshift	1430	515	0.36	No	1	-	Yes	25
787-12	17:7577538-7577538	C (31%)	T (69%)	7	c.G743G>A	p.Arg248Gln	Missense	567	1246	0.69	No	4	Deleterious	Yes	70
849-13	17:7578553-7578553	T (59%)	C (41%)	5	c.377A>G	p.Try126Cys	Missense	680	472	0.41	No	1	Deleterious	Yes	80
862-13	17:7578461-7578461	C (45%)	A (55%)	5	c.469G>T	p.Val157Phe	Missense	1333	1619	0.55	No	1	Deleterious	Yes	50
879-13	17:7577539-7577539	G (41%)	A (59%)	7	c.742C>T	p.Arg248Trp	Missense	834	1184	0.59	No	2	Deleterious	Yes	50
880-13	17:7577557-7577590	-	GGAACTGTTACACATGTAGTTGTAGTGGATGGT	7	c.723_724dupACCATCCACTACAACTACATGTGTAACAGTTCC	p.Ser241_Cys242ThrIleHisTryAsnTyrMetCysAsnSerSerCys	In-frame Insertion	N/A	N/A	N/A	No	1	-	No	10
938-13	17:7577114-7577114	C (74%)	A (26%)	8	c.824G>T	p.Cys275Phe	Missense	1815	652	0.26	No	1	Deleterious	Yes	60
943-13	17:7578407-7578407	G (55%)	C (45%)	5	c.523C>G	p.Arg175Cys	Missense	1152	936	0.45	No	1	Deleterious	Yes	40

949-13	17:7579536-7579536	C (8%)	A (92%)	4	c.151G>T	p.Glu51*	Nonsense	111	1302	0.92	Loss of WT	1	NA	Yes	80
958-13	17:7578530-7578530	A (70%)	G (30%)	5	c.400T>C	p.Phe134Leu	Missense	1081	467	0.30	No	1	Deleterious	Yes	30
1001-14	17:7578530-7578530	A (14%)	C (86%)	5	c.400T>G	p.Phe134Val	Missense	343	2129	0.86	Loss of WT	1	Deleterious	Yes	80
1004-14	17:7577121-7577121	G (11%)	A (89%)	8	c.817C>T	p.Arg273Cys	Missense	164	1394	0.89	Loss of WT	1	Deleterious	Yes	90
966-14	17:7577120-7577120	C (60%)	T (40%)	8	c.818G>A	p.Arg273His	Missense	980	651	0.40	No	1	Deleterious	Yes	30
969-14	17:7578454-7578407	G (20%)	A (80%)	5	c.476C>T	p.Ala159Val	Missense	534	2118	0.80	Loss of WT	1	Deleterious	Yes	80
985-14	17:7577058-7577058	C (35%)	A (65%)	8	c.880G>T	p.Glu294*	Nonsense	519	978	0.65	No	2	NA	Yes	20

b)

Sample ID	Genomic position (chr:start-end)	Reference allele (%)	Variant allele (%)	Exon	cDNA change	Protein effect	Mutation type	Tumor reference allele read count	Tumor variant allele read count	Tumor variant allele ratio	LOH ≥70%	No. of cases with variant	Functional classification (SIFT)	Database Presence (IARC)	Percent Tumour composition
OV202	17:7577559-7577559	G (1%)	A (99%)	7	c.722C>T	p.Ser241Phe	Missense	14	2257	0.99	Loss of WT	Not applicable	Deleterious	Yes	NA
OV207	17:7577120-7577120	C (1%)	T (99%)	8	c.818G>A	p.Arg273His	Missense	19	2139	0.99	Loss of WT	Not applicable	Deleterious	Yes	NA
OV167	17:7577046-7577046	C (1%)	A (99%)	8	c.892G>T	p.Glu298*	Nonsense	14	3026	0.99	Loss of WT	Not applicable	NA	Yes	NA

Supplementary Table S2. p53 Immunohistochemical staining of **(a)** 72 high-grade serous ovarian cancer samples and **(b)** 4 low-grade serous ovarian cancer samples.

a)

**Sample ID**	**% cells stained positive for p53**	**IHC STATUS**	***TP53* Status**
10-04	2	Low	Frameshift
14-04	100	High	Missense
18-04	100	High	Missense
40-05	100	High	Splice
47-05	100	High	Missense
64-05	100	High	Missense
74-05	2	Low	Frameshift
76-05	100	High	Missense
105-06	100	High	Missense
106-06	100	High	Missense
109-06	100	High	Nonsense
94-06	100	High	Missense
99-06	100	High	Missense
123-07	100	High	Missense
133-07	100	High	Missense
156-07	100	High	Missense
157-07	100	High	Missense
198-08	4	Low	Nonsense
206-08	100	High	Missense
230-08	0	Low	Frameshift
237-08	100	High	Missense
309-09	100	High	Nonsense

337-09	100	High	Missense
353-09	55	Intermediate	Nonsense
381-09	0	Low	Splice
416-09	1	Low	Nonsense
427-09	65	Intermediate	Wild-type
466-10	90	High	Nonsense
471-10	100	High	Missense
472-10	100	High	Missense
490-10	100	High	Frameshift
493-10	100	High	Wild-type
497-10	2	Low	Nonsense
521-10	2	Low	Splice
531-10	100	High	Missense
537-10	100	High	Missense
543-10	100	High	Missense
565-10	85	High	Splice
568-10	100	High	Missense
586-11	0	Low	Frameshift
612-11	100	High	Missense
614-11	100	High	Missense
630-11	85	High	Nonsense
631-11	100	High	Missense
634-11	100	High	Missense
638-11	0	Low	Frameshift
651-11	0	Low	Nonsense
666-11	100	High	Missense

676-11	0	Low	Splice
679-11	100	High	Missense
694-11	95	High	Missense
695-11	0	Low	Wild-type
702-11	100	High	Missense
711-11	100	High	Missense
764-12	0	Low	Frameshift
767-12	0	Low	Frameshift
778-12	100	High	Frameshift
787-12	100	High	Missense
849-13	100	High	Missense
862-13	100	High	Missense
879-13	100	High	Missense
880-13	100	High	In-frame Insertion
881-13	40	Intermediate	Wild-type
938-13	80	High	Missense
943-13	100	High	Missense
949-13	0	Low	Nonsense
958-13	100	High	Missense
1001-14	100	High	Missense
1004-14	100	High	Missense
966-14	100	High	Missense
969-14	100	High	Missense
985-14	1	Low	Nonsense

b)

Sample ID	% cells stained positive for p53	IHC STATUS	TP53 Status
544-10	8	Intermediate	Wild-type
624-11	40	Intermediate	Wild-type
909-13	65	Intermediate	Wild-type
730-12	55	Intermediate	Wild-type

Supplementary Table S3. Clinical data for **a)** 72 high-grade serous ovarian cancer; and, **b)** 4 low-grade serous ovarian cancer.

a)

Sample ID	Age	Type	Site	Grade	Stage	Status at last Follow Up (0-Alive, 1-Dead)	Duration of follow-up (months)
10-04	58	Serous adenocarcinoma	Ovary	High	3C	1	34
14-04	86	Serous adenocarcinoma	Ovary	High	1C	1	18
18-04	57	Serous adenocarcinoma	Fallopian Tube	High	3B	1	77
40-05	62	Serous adenocarcinoma	Uterus	High	3A	0	0
47-05	60	Serous adenocarcinoma	Ovary	High	2B	1	56
64-05	68	Carcinosarcoma	Ovary	High	2A	1	10
74-05	45	Serous adenocarcinoma	Ovary	High	2C	0	62
76-05	60	Serous adenocarcinoma	Ovary	High	3C	1	44
105-06	77	Serous adenocarcinoma	Ovary	High	3B	1	23
106-06	82	Serous adenocarcinoma	Ovary	High	3C	0	89
109-06	68	Serous adenocarcinoma	Ovary	High	3C	1	36
94-06	63	Serous adenocarcinoma	Peritoneum	High	3C	1	23
99-06	79	Serous adenocarcinoma	Ovary	High	3C	0	85
123-07	60	Serous adenocarcinoma	Peritoneum	High	3C	1	1
133-07	59	Serous adenocarcinoma	Ovary	High	3C	0	80
156-07	73	Serous adenocarcinoma	Peritoneum	High	3C	0	75
157-07	50	Serous adenocarcinoma	Ovary	High	3B	1	47
198-08	77	Serous adenocarcinoma	Ovary	High	3C	1	14
206-08	73	Serous adenocarcinoma	Ovary	High	3C	0	73
230-08	66	Serous adenocarcinoma	Ovary	High	3C	0	70

237-08	63	Serous adenocarcinoma	Ovary	High	3C	0	62
309-09	56	Papillary serous carcinoma	Ovary	High	3b	0	17
337-09	38	Serous adenocarcinoma	Peritoneum	High	3B	1	22
353-09	62	Serous carcinoma	Ovary	High	3B	1	9
381-09	55	Serous adenocarcinoma	Ovary	High	2A	1	33
416-09	62	Serous adenocarcinoma	Fallopian Tube	High	3B	1	7
427-09	30	Serous adenocarcinoma	Ovary	High	3B	0	8
466-10	79	Serous adenocarcinoma	Ovary	High	3C	1	32
471-10	82	Serous carcinoma	Ovary	High	3c		
472-10	57	Serous adenocarcinoma	Ovary	High	3B	1	18
490-10	64	Serous adenocarcinoma	Ovary	High	3C	1	37
493-10	68	Serous adenocarcinoma	Ovary	High	3B	1	33
497-10	43	Serous adenocarcinoma	Ovary	High	3C	0	29
521-10	36	Serous adenocarcinoma	Fallopian Tube	High	3C	0	45
531-10	77	Endometrioid adenocarcinoma	Ovary	High	2C	1	33
537-10	79	Serous adenocarcinoma	Ovary	High	3C	1	34
543-10	46	Serous adenocarcinomainoma	Fallopian tube	High	2C	0	42
565-10	61	Serous adenocarcinoma	Ovary	High	3B	0	29
568-10	80	Serous carcinoma	Ovary	High	3b	1	1
586-11	51	Serous adenocarcinoma	Ovary	High	3C	0	38
612-11	54	Serous adenocarcinoma	Ovary	High	3C	0	31
614-11	76	Serous adenocarcinoma	Ovary	High	1A	0	37
630-11	70	Serous adenocarcinoma	Fallopian Tube	High	3C	0	35

631-11	61	Serous adenocarcinoma	Ovary	High	3C	0	36
634-11	52	Serous papillary carcinoma	Ovary	High	3C	1	36
638-11	63	Serous carcinoma	Fallopian tube	High	1C		
651-11	62	Serous carcinoma	Fallopian tube	High	1A	0	35
666-11	46	Serous adenocarcinoma	Fallopian Tube	High	2A	0	34
676-11	52	Serous adenocarcinoma	Fallopian Tube	High	3C	1	16
679-11	60	Serous adenocarcinoma	Fallopian Tube	High	3C	0	31
694-11	56	Serous adenocarcinoma	Ovary	High	3C	0	26
695-11	68	Serous adenocarcinoma	Ovary	High	2B	0	23
702-11	81	Serous adenocarcinoma	Fallopian Tube	High	2C	1	3
711-11	62	Serous adenocarcinoma	Fallopian Tube	High	3C	1	15
764-12	67	Serous adenocarcinoma	Ovary	High	3C	0	3
767-12	82	Serous adenocarcinoma	Ovary	High	2B	0	24
778-12	48	Serous adenocarcinoma	Fallopian Tube	High	3C	0	22
787-12	34	Serous adenocarcinoma	Ovary	High	3B	0	24
849-13	56	Serous carcinoma.	Ovary	High	3C	0	20
862-13	72	Serous carcinoma	Ovary	High	3C	0	15
879-13	83	Serous carcinoma	Fallopian tube	High	1A	0	16
880-13	62	Serous carcinoma	Ovary	High	3C	1	12
881-13	73	Serous carcinoma	Ovary	High	3C	0	16

938-13	80	Serous carcinoma	Ovary	High	3C	0	11
943-13	89	Serous carcinoma	Ovary	High	3C		
949-13	60	Serous carcinoma	Ovary	High	3C	0	0
958-13	61	Serous carcinoma	Ovary	High	3C	0	9
1001-14	60	Serous carcinoma	Ovary	High	3C	0	0
1004-14	55	Serous carcinoma	Ovary	High	3C	0	0
966-14	76	Serous carcinoma	Ovary	High	3C	0	0
969-14	76	Serous carcinoma	Ovary	High	3C	0	5
985-14	57	Serous carcinoma	Ovary	High	3C	0	5

b)

**Sample ID**	**Age**	**Type**	**Site**	**Grade**	**Stage**	**Status at last Follow Up (0-Alive, 1-Dead)**	**Duration of follow-up (months)**
544-10	61	Serous border tumour	Ovary	Low	1C	0	35
624-11	78	Serous border tumour	Ovary	Low	1A	0	11
909-13	69	Serous adenocarcinoma	Ovary	Low	1A	0	11
730-12	47	Serous adenocarcinoma	Ovary	Low	3C	0	26

Supplementary Table S4. Cell line typing for OV207

**Marker**	**OV207**
D8S1179	13
D21S11	31.2
D7S820	8
CSF1PO	11
D3S1358	14,18
TH01	9.3
D13S317	12
D16S539	13
D2S1338	17,24
D19S433	13,14
vWA	15,16
TPOX	8
D18S51	12
Amel	X
D5S818	11,12
FGA	20

Cell typing was performed by CellBank Australia (Westmead NSW 2145, Australia) using the AmpFl STR Identifiler PCR Amplification Kit (Applied Biosystems), a 16 loci (15 STR loci plus Amelogenin) STR multiplex kit. To our knowledge, a profile for OV207 has not previously been available.

Supplementary Table S5. *TP53* primer pairs used from the IARC *TP53* Database for Sanger sequencing.

**Exon**	**Product size (bp)**	**Primer**	**5'-3'**
Exon 4	413	Forward	TGAGGACCTGGTCCTCTGAC
		Reverse	AGAGGAATCCCAAAGTTCCA
Exons 5 and 6	467	Forward	TGTTCACTTGTGCCCTGACT
		Reverse	TTAACCCCTCCTCCCAGAGA
Exon 7	237	Forward	CTTGCCACAGGTCTCCCCAA
		Reverse	AGGGGTCAGAGGCAAGCAGA
Exons 8 and 9	445	Forward	TTGGGAGTAGATGGAGCCT
		Reverse	AGTGTTAGACTGGAAACTTT
Exon 10	260	Forward	CAATTGTAACTTGAACCATC
		Reverse	GGATGAGAATGGAATCCTAT
Exon 11	245	Forward	AGACCCTCTCACTCATGTGA
		Reverse	TGACGCACACCTATTGCAAG

SUPPLEMENTARY FIGURE LEGENDS

Supplementary Figure S1. Kaplan-Meir analyses comparing overall survival with ***a)*** *TP53* mutation type (missense, frameshift, splice, nonsense or wild-type (WT)), ***b)*** missense versus all other *TP53* mutations (excluding the single insertion duplication mutation), ***c)*** *TP53* mutations located within the p53 DNA binding domain (DBD) versus outside of this domain, and ***d)*** p53 expression levels determined by p53 immunohistochemistry; ‘Low’ (L), ‘Intermediate’ (I) or ‘High’ (H).

Supplementary Figure S2. Additional representative images showing p53 IHC category, percent positive p53 nuclei and *TP53* mutation status.

Supplementary Figure S3. Representative images showing p53 staining patterns of wild-type *TP53* tumours. ^LGSC; *non-neoplastic, infiltrating lymphocytes staining blue.

Supplementary Figure S4. Representative images of cell pellets (p53 null SK-OV-3, wild-type *TP53* A2780, nonsense *TP53* mutant OV167 and *TP53* missense mutant OVCAR-3) showing p53 staining patterns.

Supplementary Figure S5. Massively parallel sequencing analysis pipeline. Fresh frozen tissue was collected from women with ovarian cancer by Kolling Institute Gynaecological Tumour Bank staff. DNA was extracted and *TP53* analyzed using the Fluidigm Access Array™ system followed by MPS using a MiSeq platform (Illumina). Raw results were processed using a bioinformatics pipeline. Analysis was performed using ANNOVAR. Variants were filtered against the 1000 genomes to exclude common SNPs. SIFT scores were used to remove all tolerated mutations and *TP53* mutations were visualised using IGV.

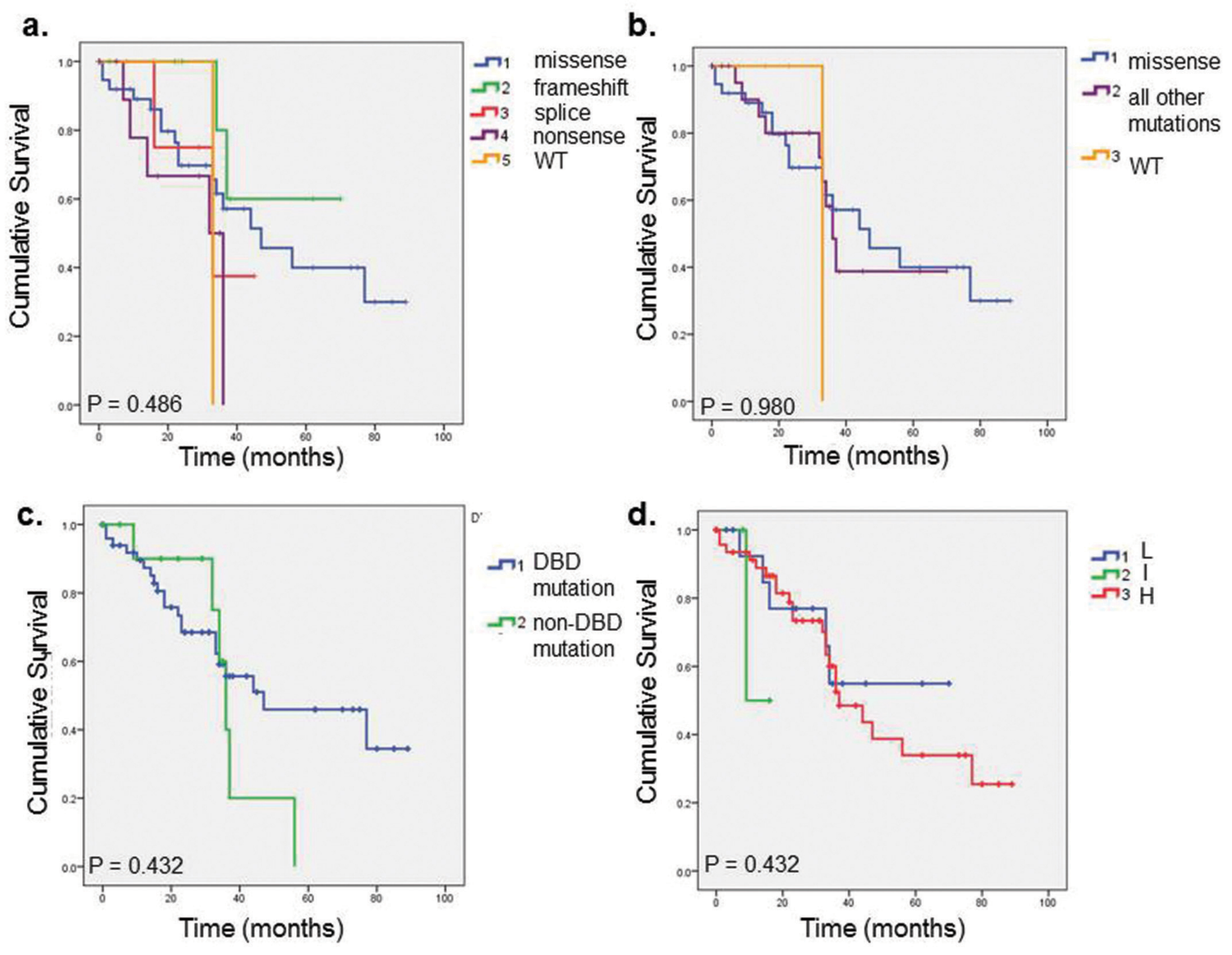

a.
Cumulative Survival
Time (months)
missense
frameshift
splice
nonsense
WT
P = 0.486
b.
missense
all other mutations
WT
P = 0.980
c.
DBD mutation
non-DBD mutation
P = 0.432
d.
L
I
H
P = 0.432

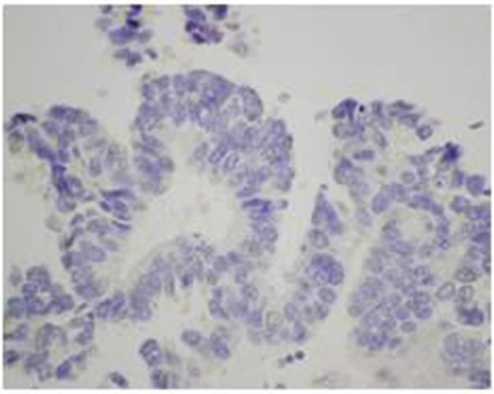

#767; 0%
p.Ala161Leufs*17

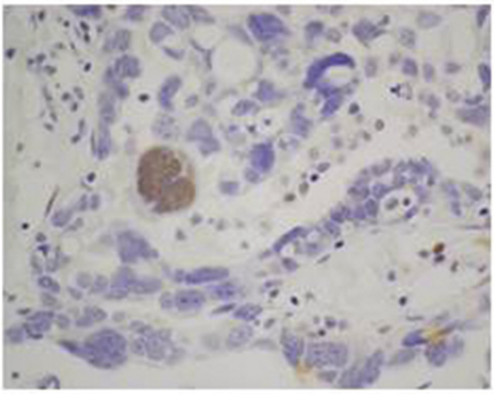

#198; 4%
p.Glu294*

LOW (≤ 5%)

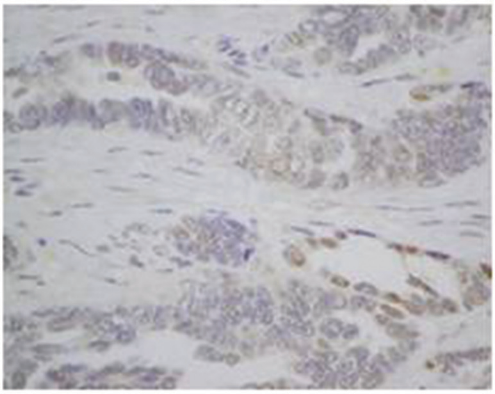

#881; 40%
TP53 wild-type

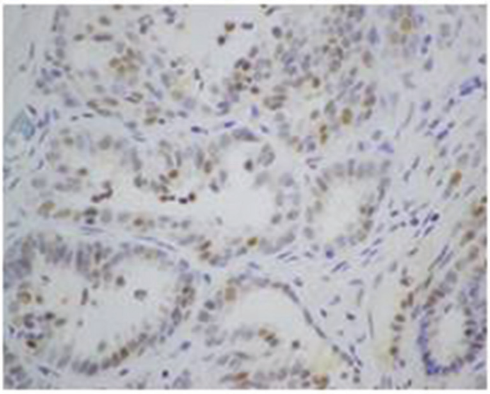

#909; 65%
TP53 wild-type

INTERMEDIATE (>5%, <70%)

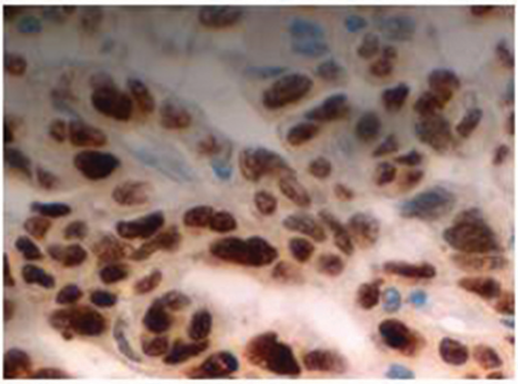

#938; 80%
p.Cys275Phe

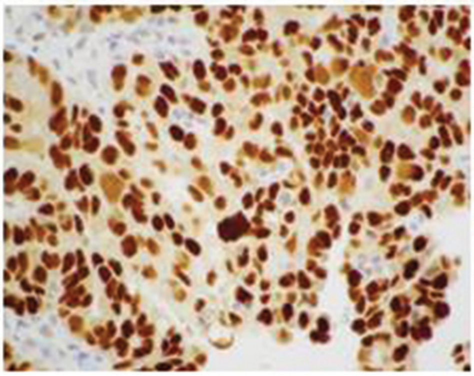

#880; 100%*

*p.Ser241_Cys242ThrIleHisTryAsnTyrMetCysAsnSerSerCys

HIGH (≥70%)

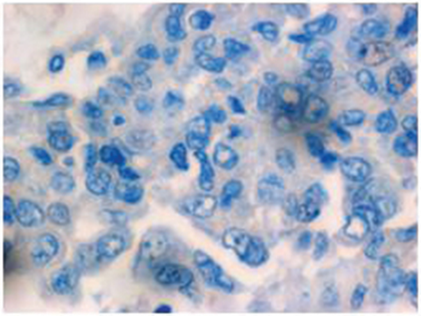

#695; 0%

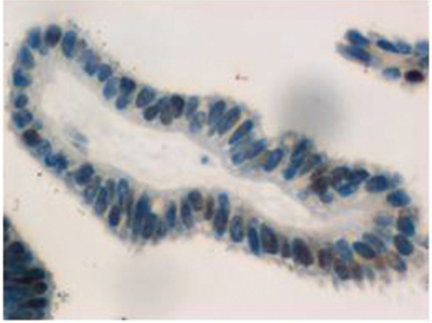

#544; 8%^

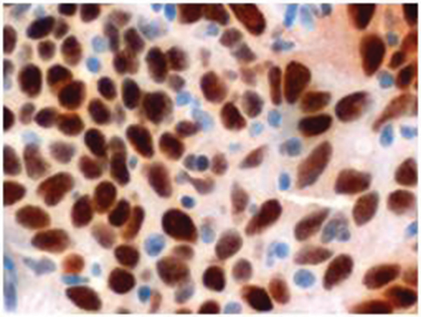

#493; 100%*

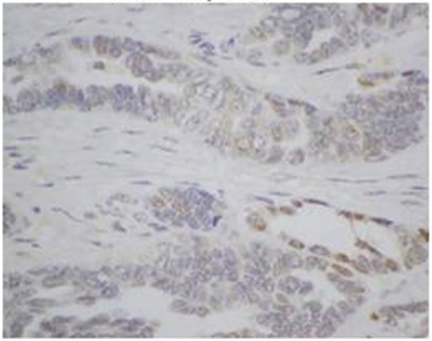

#881; 40%

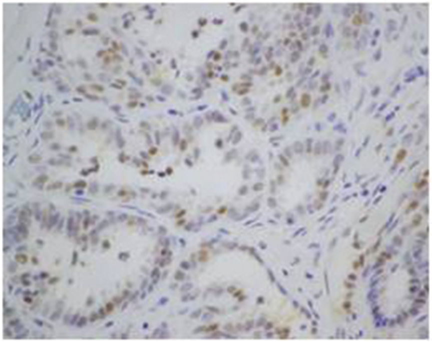

#909; 65%^

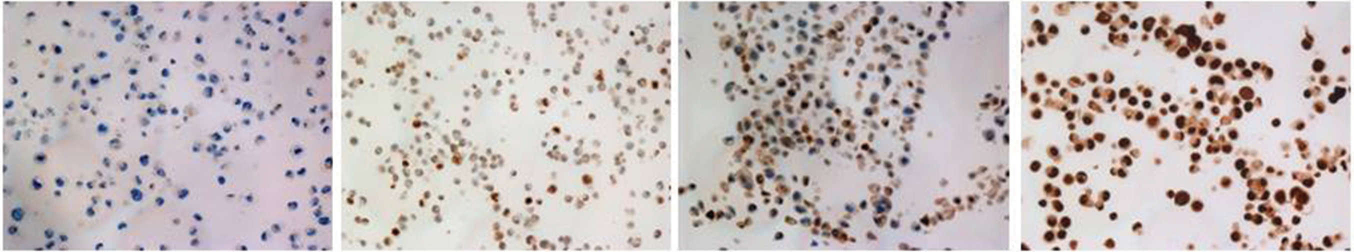

SK-OV-3
(p53 null)

A2780
TP53 wild-type

OV167
c.892G>T
p.Glu298*

OVCAR-3
c.743G>A
R248Q

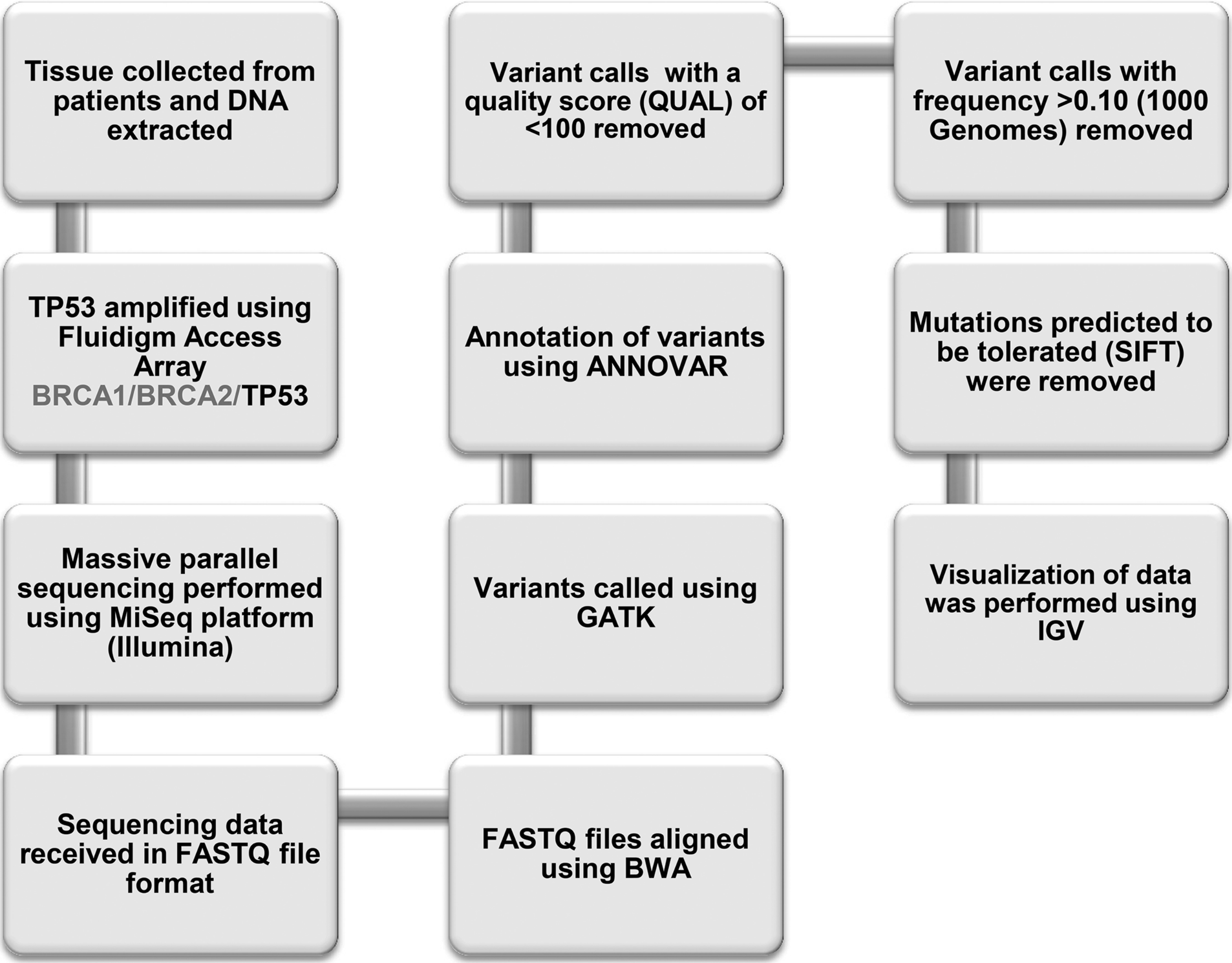
Tissue collected from patients and DNA extracted
TP53 amplified using Fluidigm Access Array BRCA1/BRCA2/TP53
Massive parallel sequencing performed using MiSeq platform (Illumina)
Sequencing data received in FASTQ file format
FASTQ files aligned using BWA
Variants called using GATK
Annotation of variants using ANNOVAR
Variant calls with a quality score (QUAL) of <100 removed
Variant calls with frequency >0.10 (1000 Genomes) removed
Mutations predicted to be tolerated (SIFT) were removed
Visualization of data was performed using IGV
